# Combined effects of cigarette smoking, gene polymorphisms and methylations of tumor suppressor genes on non small cell lung cancer: a hospital-based case-control study in China

**DOI:** 10.1186/1471-2407-10-422

**Published:** 2010-08-12

**Authors:** Yongtang Jin, Heyun Xu, Chunye zhang, Yunming Kong, Yong Hou, Yingchun Xu, Shaoli Xue

**Affiliations:** 1 Department of Environmental Medicine or Institute of Environmental Medicine, Zhejiang University School of Medicine, Zhejiang University, 310058 Hangzhou, China; 2 Department of Cardiothoracic Surgery, Sir Run Run Shaw Hospital, Zhejiang University School of Medicine, Zhejiang University, Hangzhou, 310058 China; 3 School of Public Health, Anhui Medical University, 230032 Hefei, China; 4 Institute of Pharmacology, Zhejiang University School of Pharmacology, Zhejiang University, Hangzhou, 310058 China; 5 Department of Biotechnology, Anhui Medical University, 230032 Hefei, China

## Abstract

**Background:**

Cigarette smoking is the most established risk factor, and genetic variants and/or gene promoter methylations are also considered to play an essential role in development of lung cancer, but the pathogenesis of lung cancer is still unclear.

**Methods:**

We collected the data of 150 cases and 150 age-matched and sex-matched controls on a Hospital-Based Case-Control Study in China. Face to face interviews were conducted using a standardized questionnaire. Gene polymorphism and methylation status were measured by RFLP-PCR and MSP, respectively. Logistic regressive model was used to estimate the odds ratios (OR) for different levels of exposure.

**Results:**

After adjusted age and other potential confounding factors, smoking was still main risk factor and significantly increased 3.70-fold greater risk of NSCLC as compared with nonsmokers, and the ORs across increasing levels of pack years were 1, 3.54, 3.65 and 7.76, which the general dose-response trend was confirmed. Our striking findings were that the risk increased 5.16, 8.28 and 4.10-fold, respectively, for NSCLC with promoter hypermethylation of the *p16*, *DAPK *or *RARβ *gene in smokers with *CYP1A1 *variants, and the higher risk significantly increased in smokers with null *GSTM1 *and the OR was 17.84 for NSCLC with *p16 *promoter hypermethylation, 17.41 for *DAPK*, and 8.18 for *RARβ *in smokers with null *GSTM1 *compared with controls (all p < 0.01).

**Conclusion:**

Our study suggests the strong combined effects of cigarette smoke, *CYP1A1 *and *GSTM1 *Polymorphisms, hypermethylations of *p16*, *DAPK *and *RARβ *promoters in NSCLC, implying complex pathogenesis of NSCLC should be given top priority in future research.

## Background

Lung cancer kills over one million people each year all over the world, and it is a major public health problem as the leading cause of cancer death in men and second leading cause in women [1]. The two major forms of lung cancer are non-small cell lung cancer (NSCLC, about 85% of all lung cancer) which includes squamous cell carcinoma, adenocarcinoma and large cell carcinoma, and small-cell lung cancer (SCLC, about 15%)[2]. Lung cancer mortality has increased rapidly during recent years in Asian countries as the use of tobacco products is increasing [3]. About 80-90% of lung cancers are attributable to cigarette smoking, and an estimated 20% of all lung cancers are caused by a combination of environmental and/or genetic factors [4], but inter-individual differences in carcinogen metabolism may play an essential role in the initiation and progression of this environmental cancer and affect individual susceptibility to lung cancer [5,6]. Cigarette tobacco contains a variety of carcinogens, such as polycyclic aromatic hydrocarbons(PAHs), N-nitrosoamines, and aromatic heterocyclic amines [7]. PAHs are metabolized to reactive DNA binding diols epoxides by phase I (e.g. *CYP1A1*) and detoxified by phase II (e.g. *GSTM1*) before targeting DNA. It is possible that individual variations in metabolic activities in each phase or both phases of metabolism coordinately modulate the clearance of DNA [8]. Many studies have reported that polymorphism in *CYP1A1 *as well as in *GSTM1*, or combination effect of both, have been associated with different types of cancer risk including human lung cancer [9].

It is now recognized that not only genetic mechanisms, such as gross chromosomal alterations or single nucleotide mutations, but also aberrant DNA methylation provides one or both of the two hits postulated in Knudson's two hit hypothesis for the inactivation of tumor suppressor genes. Many studies have indicated that aberrant methylation of the promoter causes transcriptional silencing of some important suppressor genes, such as cell cycle gene *p16*, apoptosis gene *DAPK*, cell differentiation and proliferation gene *RARβ*, DNA repair gene *MGMT*, and this has been implicated in the carcinogenic process in human lung cancer [4]. Furthermore, methylation has been described as an early event in lung tumorigenesis and variation in methylation status has been associated with cigarette smoke exposure [10,11]. In addition, only a relative small study has examined the relationship between polymorphisms in *XRCC1*, *GSTM1*, *GSTP1*, *NQO1*, and *MPO *and aberrant methylation of *p16*, *RARβ *and *MGMT *in lung cancer [6]. Those result suggested that *GSTP1 *and *NQO1 *variations increased the risk of *MGMT *methylation, and the possibility of *p16 *and *RARβ *methylations was increased for *XRCC1 *and *MPO *gene polymorphisms, indicating the interactions between gene polymorphisms and aberrant methylation of tumor suppress genes.

Above facts led us hypothesize that major metabolic enzyme gene genetic polymorphisms and environmental factors, such as cigarette smoking and diet habits, may interact during the hypermethylations of tumor suppressor gene (TSG) promoters in the carcinogenesis of NSCLC. So, the present study have mainly investigated the association between cigarette smoking, polymorphisms of CYP1A1 and GSTM1 genes, hypermethylations of *p16*, *DAPK *and *RARβ *gene promoters in NSCLC.

## Methods

### Study population

150 individuals with NSCLC and 150 age-matched (within 3 years) and sex-matched controls were enrolled in this study from June 2005 to December 2007 at 1^st^Affiliated hospital of Anhui Medical University, China. Of patients with lung cancer, histological subtype including 83 squamous cell carcinomas, 33 adenocarcinomas and 34 mixed types of both were confirmed. 57 patients had stage I disease, 64 patients had stage II disease, 20 patients had stage III A disease, 5 patients had stage III B disease, and 4 patients had stage IV disease. Controls were selected from patients newly diagnosed with diseases other than cancer and chronic respiratory diseases or from individuals receiving routine medical examinations at the same hospitals. There were no significant difference of mean age between cases and controls (59.81 ± 9.18 *vs *59.91 ± 8.71 years). There were 125 males and 25 females in cases or controls group. This study was approved by the Ethical Committee of Anhui Medical University and conducted in accordance with the recommendations outlined in the Declaration of Helsinki, and all subjects provided written informed consent.

### Exposure to environmental factors

Trained interviewers used a structured questionnaire to interview each subject face to face when the subjects agreed to take part in this study and underwent medical examination. The questionnaire mainly included questions on demographic factors, smoking history (duration and daily consumption of cigarettes), consumption of alcohol, tea drinking and dietary factors (i.e. intake of peppery and/or fruit), family history of cancer in first relatives (i.e., parents, siblings and offspring), and clinical features of lung cancer and complete medical history. Smoking habit was defined as smoking more than 1 cigarette a day for at least 1 year, or more than 360 cigarettes a year. Pack years were calculated by multiplying the number of packs of cigarettes smoked a day by the number of years the person had smoked. Alcohol habit was defined as drinking more than twice a week, consumption of more than 50 ml of heavy liquor or 500 ml of beer on each occasion. Tea habit was defined as drinking tea at least one time a day for at least 1 year. The servings of peppery or fruit was defined to intake more than twice a week.

### DNA extraction and genotyping

Cases and controls were asked to provide 5 ml peripheral venous blood. This was separated in two aliquots of 1 ml serum and in two aliquots of buffy coats and stored at -20°C. Genomic DNA was extracted from the buffy coats using QIA Gen Blood Kit according to the manufacture's instructions (Qiagen). A DyNA Quant 200 fluormeter (Hoefer, San Francisco, CA) was used to test DNA concentration. *CYP1A1 *(MspI) polymorphism and *GSTM1 *deletion were measured by RFLP-PCR on ABI 9600 Thermal Cycler. Table 1 showed the main parameters of PCR.

**Table 1:** Summary of primer sequences, annealing temperatures and PCR product sizes used for *CYP1A1 *(MspI) and *GSTM1*

Gene	Primer	°C	bp
*CYP1A1 *(MspI)	Forward 5'-CAGTGAAGAGGTGTAGCCGCT-3'	60	340
	Reverse 5'-TAGGAGTCTTGTCTCATGCCT-3'		
*GSTM1*	Forward 5'-GAACTCCCTGAAAAGCTAAAGC-3'	59	215
	Reverse 5'-GTTGGGCTCAAATATACGGTG G-3'		
β-Globin (reference)	Forward 5'-CAACTTCATCCACGTTCACC-3'	59	268
	Reverse 5'-GAAGAGCCAAGGACAGGTA-3'		

### Methylaiton-specific PCR for p16, DAPK and RARβ promoters

Primary tumor and corresponding nonmalignant lung tissue samples (n = 150, separately) were obtained from NSCLC patients who had been treated with curative resectional surgery in 1^st ^Affiliated hospital of Anhui Medical University between June 2005 and December 2007. Those samples were snap-frozen in liquid nitrogen and stored at -70°C until genomic DNA preparation. Genomic DNA was extracted using QIA tissue kitaccording to the manufacturer's instructions (Qiagen). A DyNA Quant 200 fluorometer (Hoefer, San Francisco, CA) was used to measure DNA concentration.

Methylation status of the promoter region of the *p16*, *DAPK *and/or *RARβ *was determined by MSP described by Zochbauer-Muller et al. [12]. Two sets of primers were designed, one specific for DNA methylated at the promoter region of each gene and the other specific for unmethylated DNA (Table 2). Amplification was carried out on ABI 9600 Thermal Cycler.

**Table 2:** Summary of primer sequences*, annealing temperatures, and PCR product sizes used for MSP

Gene	Forward primer (5' - 3')	Reverse primer (3' - 5')	Tm (°C)	Size (bp)
*p16*	M: TTATTAGAGGGTGGGGCGGATCGC**	M: GACCCCGAACCGCGACCGTAA	60	150
	U: TTATTAGAGGGTGGGGTGGATTGT	U: CAACCCCAAACCACAACCATAA	60	151
*DAPK*	M: GGATAGTCGGATCGAGTTAACGTC	M: CCCTCCCAAACGCCGA	59	98
	U: GGAGGATAGTTGGATTGAGTTAATGTT	U: CAAATCCCTCCCAAACACCAA	59	106
*RARβ*	M: TCGAGAACGCGAGCGATTCG	M: GACCAATCCAACCGAAACGA	56	146
	U: TTGAGAATGTGAGTGATTTGA	U: AACCAATCCAACCAAAACAA	56	146

* Reference for primer sequences: Ref. [13].

** M. methylated-specific primers: U. unmethylated-specific primers.

### Data analysis

To determine the association between each of the test genes and lung cancer, the homozygous (AA or aa genotype) and heterozygous (Aa genotype) states of the variants were first analyzed as categorical variables, and then reanalyzed as dichotomized variables grouped by the risk genotype (i.e., 0 for the wild type homozygous, and 1 for the other genotypes combined). To evaluate the effects of combined genotypes, environmental factors either together or separately, subjects were categorized into homozygous wild type, and possession of one or more of the risk genotypes (heterozygous + homozygous for the variant). Compared with the wild type genotype, the odds ratio (OR) and 95% confidence interval (CI) of the various genotypes was calculated for lung cancer risks in univariate analysis model. Multivariate logistic regression was conducted to estimate the relationship between smoking, polymorphisms of metabolic enzyme genes and methylation inactivate of tumor suppressor genes in NSCLC after adjusted the potential confound factors. SAS software (version 9.1; SAS Institute, Inc.) was used for statistical analysis, using the *x*^*2 *^and Fisher's exact test for differences between groups and *t *tests between means. All tests were two-sided, and a *p *value of <0.05 for any test or models was considered statistically significant.

## Results

The ORs of major risk factors among cased and controls are shown in Table 3. After adjusting for potential confounders, there were no significant differences between the cases and controls in alcohol habit, tea habit, dust exposure (≥1 month/year), toxin exposure (≥1 month/year), and the family history of lung cancer among first relatives of patients. Genotype frequencies for *CYP1A1 *and *GSTM1 *are calculated, which these distributions are consistent with the Hardy-Weinberg equilibrium model. In the control group, the allele frequency for MspI was 0.30 (a), whereas that for lung cancer group was 0.29. A non-significant difference was observed between cases and controls. In addition, 53% of controls and 63% of cases were homozygous for null variant allele of *GSTM1*. No significant associations between the variants of *CYP1A1 *or *GSTM1 *and lung cancer. However, significant associations were also found between lung cancer and the follow variables: smoking habit, pack years, peppery (servings, > 2 times/week), and fruit (servings, > 2 times/week) (Table 3).

**Table 3:** ORs of major risk factors for non-small cell lung cancer.

Factor	Crude risk			Adjusted risk		
		
	OR	95% CI	p-value	**OR**^**a**^	95% CI	p-value
Smoking habit(yes/no)	2.21	1.35-3.3.63	<0.01	3.70	1.49-9.19	p < 0.01
Pack years <20	1.00	-	-	1.00	-	-
20-30	3.27	1.43-7.49	<0.01	3.54	1.11-11.26	p < 0.05
30-40	3.53	1.72-7.26	<0.01	3.65	1.27-10.50	p < 0.05
≥40	7.54	3.95-14.41	<0.01	7.76	3.00-20.11	p < 0.01
*x*^*2 *^test for trend	p < 0.001			p < 0.001		
Alcohol habit(yes/no)	1.46	0.92-2.30	>0.05	1.72	0.83-3.53	p > 0.05
Tea habit(yes/no)	1.14	0.69-1.89	>0.05	0.92	0.41-2.09	p > 0.05
Peppery (servings, > 2 times/week, yes/no)	0.61	0.38-0.98	<0.05	0.35	0.16-0.76	p < 0.01
Fruit (servings, > 2 times/week, yes/no)	0.28	0.14-0.58	<0.01	0.16	0.06-0.43	p < 0.01
Dust exposure^b ^(yes/no)	3.55	2.08-6.04	<0.01	2.06	0.93-4.57	p > 0.05
Toxin exposure^b ^(yes/no)	1.31	0.79-2.18	>0.05	0.68	0.30-1.56	p > 0.05
Lung cancer in firstdegree relatives (yes/no)	1.35	0.63-2.88	>0.05	1.44	0.41-5.06	p > 0.05
*CYP1A1*((Aa + aa)/AA)^c^	1.03	0.65-1.62	>0.05	0.83	0.42-1.65	p > 0.05
*GSTM1*(null/power)^d^	1.55	0.98-2.46	>0.05	1.13	0.57-2.24	p > 0.05

^a ^adjusted for sex, age, alcohol habit, tea habit, smoking habit and history of lung cancer in first-degree relatives.

^b ^cumulative exposure ≥3 months/year or continuous exposure ≥1 months/year.

^c ^AA = homogeneity wild genotype, Aa = heterogeneity genotype, aa = homogeneity variant genotype

^d ^- = null type, + = power

This study confirmed smoking was the main risk factor of lung cancer, and increased 3.70 times greater risk of NSCLC compared with nonsmoker. Further, the OR of NSCLC increased with higher categories of total smoking pack year, from 3.54 in the second category to 7.76 in the fourth category (Table 3). ORs of the three higher categories were all statistically significant. After adjustment for the potential confounding factors in the multivariate analysis models, ORs in each category of smoking pack years increased, and CIs became wider, but the general dose-response trend was maintained (Table 3). Interestingly, we found the preventive effects of peppery or fruit servings on lung cancer, and OR was 0.35 (95%CI, 0.16-0.76) and 0.16 (95%CI, 0.06-0.43), respectively. This study suggested non-significant association of variants of *CYP1A1 *and *GSTM1 *with NSCLC alone or in combination. However, the risk increased about 4-fold in smokers with *CYP1A1 *variants as compared with *CYP1A1 *wild homozygous non-smokers and 7-fold when smokers having null *GSTM1 *were compared with power *GSTM1 *non-smokers. These results can imply the interactions of smoking and the genetic variants of *CYP1A1 *and *GSTM1 *in NSCLC (Table 4).

**Table 4:** The interactions of smoking, *CYP1A1 *and *GSTM1 *variants in non-small cell lung cancer

Factor		Case(n)	Control(n)	Crude OR (95% CI)	**Adjusted OR**^**a **^**(95% CI)**
*CYP1A1*^b^	*GSTM1*^c^				
				
Aa+aa	-	43	39	1.63(0.81-3.31)	1.67(0.85-3.29)
	+	28	31	1.34(0.62-2.89)	1.55(0.74-3.25)
AA	-	52	40	1.93(0.97-3.84)	2.03(1.04-3.94)*
	+	27	40	1.00(reference)	1.00(reference)

Smoking habit	*CYP1A1*				
				
Yes	Aa+aa	52	39	2.37(1.10-5.15)*	3.72(1.56-8.86)**
	AA	61	48	2.26(1.07-4.78)*	3.47(1.47-8.16)**
No	Aa+aa	19	31	1.09(0.45-2.66)	1.08(0.47-2.51)
	AA	18	32	1.00(reference)	1.00(reference)

Smoking habit	*GSTM1*				
				
Yes	-	70	51	4.00(1.79-9.09)**	6.76(2.62-17.46)**
	+	43	36	3.48(1.48-8.33)**	5.93(2.26-15.59)**
No	-	25	28	2.60(1.03-6.67)*	2.84(1.17-6.92)*
	+	12	35	1.00(reference)	1.00(reference)

^a ^adjusted for sex, age, alcohol habit, tea habit, smoking habit and history of lung cancer in first-degree relatives.

^b ^AA = homogeneity wild genotype, Aa = heterogeneity genotype, aa = homogeneity variant genotype

^c ^- = null type, + = power

* p < 0.05 ** p < 0.01

We used MSP to determine the frequency of methylation of *p16*, *DAPK *and *RARβ *in 150 resected NSCLCs, which was 48.67%, 58.67% and 60.00%, respectively. In the corresponding nonmalignant lung tissues, it was seen at low frequencies for *p16 *(9.93%), *DAPK *(9.93%) and *RARβ *(17.02%). Those indicated the significant difference between lung cancer tissues and nonmalignant lung tissue in methylations of three genes. In addition, we found that at least one of these three genes had methylation in 85.33% of the tumors; 26% of the tumors had only one gene methylated, 36.67% of the tumors had two genes methylated and 22.67% of the tumors had three genes methylated. A statistically significant corrrlation was found for the methylation status between *p16 *and *DAPK *(p = 0.0006), whereas the methylation status of the other genes was independent when compared with each other. Although no association was apparent among the *CYP1A1 *or *GSTM1 *polymorphisms and *p16*, *DAPK *or *RARβ *promoter methylation, *GSTM1 *null genotype was significantly associated with at least one methylation among *p16*, *DAPK *and *RARβ *genes (OR, 1.67; 95% CI, 1.01-2.77) (no data shown).

Table 5 presents OR estimates for smoking habits, pack years, diet habits, family history of lung cancer, and polymorphisms of *CYP1A1 *and *GSTM1 *as compared with controls according to the cases with or without promoter hypermethylation of the *p16*, *DAPK *or *RARβ *gene. Obviously, smoking habits increased the risk of NSCLC with promoter hypermethylation of the *p16*, *DAPK *or *RARβ*, which OR is 4.56, 3.83, 3.11, respectively. As the amount of pack years increased, the risk of NSCLC with promoter hypermethylation of the *p16*, *DAPK *or *RARβ *gene was greater, indicating a graded positive association between both. The results may also imply the interaction between cigarette smoking and promoter hypermethylation of the *p16*, *DAPK *or *RARβ *gene in NSCLC. In addition, a possible association was found between null *GSTM1 *and NSCLC with promoter hypermethylation of the *DAPK *or *RARβ *gene, implying effect of *GSTM1 *polymorphism on the aberrant methylations of TSG in lung cancer. Of note, higher consumption of fruit was associated with lower risk of NSCLC with or without promoter hypermethylation of the *p16*, *DAPK *or *RARβ *gene (no data shown) (Table 5).

**Table 5:** The interactions between cigarette smoking, genetic variants of *CYP1A1*and *GSTM1*, and promoter hypermethylations of the *p16*, *DAPK *and *RARβ *genes in non-small cell lung cancer

Factor	OR ^a ^(95% CI)					
	
	Cases with TSG^b ^promoter hypermethylation			Cases without TSG promoter hypermethylation		
	
	p16	DAPK	RARβ	p16	DAPK	RARβ
Smoking habit (yes/no)	4.56**	3.83**	3.11**	1.34	1.23	1.45
	(2.17-9.58)	(1.99-7.38)	(1.67-5.78)	(0.76-2.37)	(0.67-2.26)	(0.77-2.71)
Pack years						
<20	1.00	1.00	1.00	1.00	1.00	1.00
	(reference)	(reference)	(reference)	(reference)	(reference)	(reference)
20-29	4.62**	6.72**	3.27*	2.60	1.23	3.27*
	(1.63-13.13)	(2.63-17.18)	(1.23-8.69)	(0.99-6.80)	(0.37-4.12)	(1.19-9.01)
30-39	5.08**	6.82**	4.27**	1.75*	1.58	2.70*
	(2.04-12.69)	(2.2.94-15.81)	(1.88-9.70)	(1.19-6.36)	(0.59-4.20)	(1.06-6.89)
≥40	14.29**	13.50**	9.50**	4.17**	4.01**	5.34**
	(6.55-31.18)	(6.31-28.89)	(4.62-19.53)	(1.96-8.88)	(1.85-8.68)	(2.40-11.88)
*x*^*2 *^test for trend	p < 0.001	p < 0.001	p < 0.001	p < 0.001	p < 0.001	p < 0.001
*CYP1A1 *((Aa+aa)/AA) ^c^	0.97	1.14	1.06	1.07	0.88	0.94
	(0.55-1.81)	(0.67-1.92)	(0.62-1.80)	(0.61-1.86)	(0.48-1.59)	(0.51-1.71)
*GSTM1 *(null/power) ^d^	1.45	1.73*	1.98**	1.67	1.33	1.10
	(0.82-2.56)	(1.01-2.99)	(1.15-3.44)	(0.96-2.94)	(0.73-2.43)	(0.60-2.00)

^a ^adjusted for sex, age, alcohol habit, tea habit, smoking habit and history of lung cancer in first-degree relatives.

^b ^TSG = tumor suppress gene including *p16*, *DAPK *and *RARβ*.

^c ^AA = homogeneity wild genotype, Aa = heterogeneity genotype, aa = homogeneity variant genotype

^d ^- = null type, + = power

* p < 0.05, **p < 0.01

Based on above results, Table 6 considers the interaction between smoking habits, polymorphisms of *CYP1A1 *and *GSTM1 *variants in NSCLC with or without promoter hypermethylations of the *p16*, *DAPK *or *RARβ *gene as compared with controls. We didn't found the interaction between *CYP1A1 *polymorphisms and *GSTM1 *variant in NSCLC with or without promoter hypermethylation of the *p16*, *DAPK *or *RARβ *gene. Nevertheless, as compared with controls, the risk increased 5.16, 8.28 and 4.10-fold, respectively, for NSCLC with promoter hypermethylation of the *p16*, *DAPK *or *RARβ *gene in smokers with *CYP1A1 *variants (Aa+aa). Strikingly, the risk strongly increased in smokers with null *GSTM1*, and the OR was 17.84 for NSCLC with *p16 *promoter hypermethylation, 17.41 for *DAPK*, and 8.18 for *RARβ *in smokers with null *GSTM1 *compared with controls. In contrast, the smokers with null *GSTM1 *have lower risk for NSCLC without TSG promoter hypermethylation. To a certain extent, these results are in agreement with a previous multiplicative model for risk combination between smoking habits and metabolic enzyme gene polymorphisms analyzed when the cases were not stratified by TSG methylation status. These results may further confirm the interactions between smoking, genetic variant of *CYP1A1 *and *GSTM1*, and promoter hypermethylation of the *p16*, *DAPK *or *RARβ *gene in NSCLC (Table 6).

**Table 6:** Interactions between cigarette smoking and the genetic variants of *CYP1A1 *and *GSTM1 *in non-small cell lung cancer with or without promoter hypermethylations of the *p16*, *DAPK *and *RARβ *genes

Factor		**OR**^**a **^**(95% CI)**					
		
		**Cases with TSG**^**b **^**promoter hypermethylation**			Cases without TSG promoter hypermethylation		
		
		p16	DAPK	RARβ	p16	DAPK	RARβ
*CYP1A1*^c^	*GSTM1*^d^						
						
Aa+aa	-	1.41	2.05	2.12*	1.96	1.18	1.03
		(0.64-3.08)	(0.94-4.47)	(0.99-4.52)	(0.84-4.59)	(0.50-2.81)	(0.41-2.56)
	+	0.97	1.48	1.12	1.88	1.19	1.61
		(0.40-2.34)	(0.63-3.47)	(0.47-2.69)	(0.76-4.61)	(0.48-2.97)	(0.66-3.94)
AA	-	1.44	2.14*	2.07	2.64*	1.69	1.75
		(0.66-3.12)	(0.99-4.63)	(0.97-4.40)	(1.16-5.99)	(0.75-3.82)	(0.76-4.03)
	+	1.00	1.00	1.00	1.00	1.00	1.00
		(reference)	(reference)	(reference)	(reference)	(reference)	(reference)
Smoking habit	*CYP1A1*						
						
Yes	Aa+aa	5.16*	8.28**	4.10**	2.73	1.58	2.82
		(1.45-18.35)	(2.38-28.75)	(1.43-11.76)	(0.97-7.64)	(0.54-4.68)	(0.87-9.16)
	AA	4.75*	6.57**	3.50*	2.60	2.16	3.29
		(1.35-16.65)	(1.91-22.64)	(1.22-10.01)	(0.95-7.11)	(0.77-6.02)	(1.07-10.11)
No	Aa+aa	0.94	0.85	0.91	1.20	1.39	1.38
		(0.24-3.68)	(0.25-2.88)	(0.31-2.70)	(0.47-3.08)	(0.52-3.72)	(0.48-3.96)
	AA	1.00	1.00	1.00	1.00	1.00	1.00
		(reference)	(reference)	(reference)	(reference)	(reference)	(reference)
Smoking habit	*GSTM1*						
						
Yes	-	17.84**	17.41**	8.18**	4.43**	2.76	4.69*
		(3.10-102.64)	(4.21-72.05)	(2.53-26.49)	(1.54-12.78)	(0.88-8.60)	(1.29-17.05)
	+	17.62**	12.29**	5.14**	3.09*	3.35*	7.10**
		(3.08-100.91)	(2.95-51.21)	(1.55-17.05)	(1.04-9.14)	(1.08-10.39)	(1.98-25.41)
No	-	6.18**	2.81	2.47	2.24	3.00*	3.68*
		(1.14-33.56)	(0.77-10.20)	(0.79-7.72)	(0.85-2.90)	(1.05-8.55)	(1.15-11.77)
	+	1.00	1.00	1.00	1.00	1.00	1.00
		(reference)	(reference)	(reference)	(reference)	(reference)	(reference)

^a ^adjusted for sex, age, alcohol habit, tea habit, smoking habit and history of lung cancer in first-degree relatives.

^b ^TSG = tumor suppress gene including *p16*, *DAPK *and *RARβ*

^c ^AA = homogeneity wild genotype, Aa = heterogeneity genotype, aa = homogeneity variant genotype

^d ^- = null type, + = power

* p < 0.05, **p < 0.01

## Discussion

Many epidemiologic studies have demonstrated cigarette smoking is the major risk factor of lung cancer [13-15], with a obvious dose-response relationship [16]. Our findings (OR = 3.70, p < 0.01) supported these results unquestionably. There are more than 4000 chemical materials in cigarette smoking, and approximately 200 may be carcinogens, such as aromatic hydrocarbons, which have proved to cause lung carcinogenesis, and increasing mortality from lung cancer is closely associated with the consumption of tobacco [14]. Although the majority of lung cancer patients are smokers, only 10-15% of all smokers will develop the disease [17], indicating environmental or genetic determinants in disease initiation, promotion and progression. Since many carcinogens require metabolic activation via phase I enzymes to enable to react with cellular macromolecules or metabolic detoxification via phase II enzymes to enable to eliminate from body, inter-individual differences in carcinogen metabolism may play a key role in environmental cancers [4,6]. The most frequently studied phase I and II enzymes include *CYP1A1 *and *GSTM1*. Studies from Japanese populations first found an association between *CYP1A1 *and polymorphisms and risk of lung cancer, with reports of >2-fold increased risk [18]. In a pooled analysis using data from 22 studies, a significant 2.4-fold increased in risk was observed in individuals carrying the MspI variant [19]. In addition, *GSTM1 *occurs in the null form in ~50% of the Caucasian population. One of the first meta-analyses showed a modest increase in lung cancer among carriers of the *GSTM1*-null genotype (OR = 1.13, 95%CI 1.04-1.25) [20]. The most recent and large meta-analysis [9] of Chinese population found that lung cancer risk for *CYP1A1 *variant was 1.34-fold (95%CI 1.08-1.67, p = 0.008) compared with the wild-type homozygous genotype, and the risk for the *GSTM1 *null genotype was 1.54-fold (95%CI 1.31-1.80, p < 0.001) as compared with the *GSTM1 *present genotype. A recent pooled analysis also suggested that genetic polymorphisms in *CYP1A1 *and *GSTM1 *are associated with lung cancer risk among Asian populations [3]. Few studies have researched gene-gene interactions in lung cancer. An early study from Japan [18] reported the combined effects of *CYP1A1 *MspI genotype and deficient *GSTM1 *in lung cancer (OR = 16.00), but only at a low-dose level of cigarette smoking. Also, another analysis indicated a possible interaction between the CYP1A1*2A allele and *GSTM1 *deletion on lung cancer risk in Caucasians [21]. However, as other studies have reported conflicting results for *CYP1A1 *and *GSTM1 *polymorphisms in lung cancer [4,6], our study found neither significant risk of lung cancer for *CYP1A1 *variants or *GSTM1 *null genotypes nor possible combination effects of *CYP1A1 *and *GSTM1 *polymorphisms in the development of lung cancer. The majority of epidemiological studies on the effects of low penetrant genes in cancer etiology have considered main effects single nucleotide polymorphisms, or gene-environment interactions and rarely gene-gene interactions, mainly duo to the lack of statistical power [22]. Most observed associations between cancer and low penetrant gene variants have been weak or very weak [21]. However, penetrance of a gene variant depends on events such as the interaction with external exposures, with the internal environment or with other factors (e.g., gene promoter methylation).

In the present study, the significant interaction between cigarette smoking and *CY1A1 *or *GSTM1 *variants is consistent with the results of previous pooled analysis that the stronger association between the *CYP1A1 *MspI or *GSTM1 *null and lung cancer was found among smokers [22], but a non significant elevated risk of interaction between *GSTM1 *null genotype and lung cancer was reported among Asian by Benhamou and co-workers [23]. Cigarette smoking is known to be causally related to BPDE-DNA adducts that is elevated in the lung tissue of smokers with *GSTM1 *null genotype, which was found to induce mutations in the hotspot codons of the p53 gene [3,24]. Thus, we speculated that the interaction between *CYP1A1 *or *GSTM1 *polymorphisms and lung cancer is related to polycyclic aromatic hydrocarbons exposure derived from smoking because polycyclic aromatic hydrocarbons are primarily metabolized by *CYP1A1 *and *GSTM1*. The greater effects observed among smokers support the smoking-related etiology of lung cancer in Chinese population.

It is now recognized that not only the inherited variation in DNA sequence (e.g. gene mutations) but also the epigenetic events, such as aberrant DNA methylatoin, both play an essential role in the origination and development of lung cancer. The most widely studied epigenetic event in relation to lung cancer included the promoter hypermethylation of *p16*, *DAPK *or *RARβ *gene [4,6]. Our findings reported the percentage for *p16*, *DAPK *or *RARβ *methylated was the 48.67%, 58.67% and 60.00% in the tumor tissues of patients with lung cancer, respectively. Those results were separately a little greater than other findings that *p16 *is methylated in ~25-41% of NSCLC, *DAPK *in 16-44% and *RARβ *in 40-43% [25,26], which the differences may mainly result from ethnic variants. The study examined the relationship between polymorphisms in *CYP1A1 *and *GSTM1 *and aberrant methylation of *p16*, *DAPK *and *RARβ *in lung cancer. It is the first to found *GSTM1 *null was associated with at least one methylation of *p16*, *DAPK *and *RARβ *gene promoters (OR = 1.67, 95% CI 1.01-2.77), supporting interaction between metabolic enzyme gene polymorphisms and hypermethylation of tumor suppressor genes in development of NSCLC [27,28]. Also, data from our unconditional logistic models is the first to show that tobacco smoke play dominant roles in NSCLC with hypermethylation of *p16*, *DAPK *or *RARβ *promoter, but not without hypermethylation of those gene promoters. As the amount of cigarette smoking increased, the risk of NSCLC with *p16*, *DAPK *or *RARβ *promoter hypermethylation increased. To our knowledge, we have first reported the interactions between smoking and polymorphisms of *CYP1A1 *and *GSTM1 *gene were significantly modified by hypermethylation of *p16*, *DAPK *or *RARβ *promoter in NSCLC, indicating the combined effects of smoking, *CYP1A1*, *GSTM1*, *p16*, *DAPK *and *RARβ *gene on development of NSCLC. The findings suggest that smoking related biological pathways leading to the development of lung cancer involve not only hypermethylations of *p16*, *DAPK *and *RARβ *promoters but also genetic polymorphisms of *CYP1A1 *and *GSTM1 *genes. Although it is unclear that environmental factors underlie the targeting of specific gene promoters for hypermethylation, the characterization of gene-environment interaction and epigenetic influences in carcinogenesis is of great importance for preventive measures such as the setting of exposure threshold values, public health campaigns and chemopreventive approaches. Those all need to be further confirmed and thoroughly studied in different populations.

This study has some strengths and limitations. This is first study on the interaction between cigarette smoking and the polymorphisms of *CYP1A1 *or *GSTM1 *for NSCLC with hypermethylations of *p16*, *DAPK *and *RARβ *promoters, which carefully controlled for important confounding factors. The selective bias was mostly controlled by the design of a hospital-based case-control study. As other case-control studies, this study raises concern about recall bias and residual confounding. Of course, the major difficult is still the inability to separate exposures to factors prior to clinical onset from exposures to factors after clinical onset.

In conclusion, this study confirmed that cigarette smoking is significantly associated with higher risk of NSCLC having hypermethylation of *p16*, *DAPK *or *RARβ *promoter, and a general dose-response trend was confirmed. A striking finding was that the interactions between smoking and polymorphism of *CYP1A1 *or *GSTM1 *gene increased significantly greater risk of NSCLC with hypermethylation of *p16*, *DAPK *or *RARβ *promoter, suggesting complex pathogenesis of NSCLC should be given top priority in future research.

## Competing interests

The authors declare that they have no competing interests.

## Authors' contributions

HX gathered clinico-pathological data. CZ, YK, YH and YX were involved in conception of the study, analysis of the data and interpretation of the results. YJ and SX designed the study and wrote the manuscript. All authors approved the final manuscript.

## Pre-publication history

The pre-publication history for this paper can be accessed here:

http://www.biomedcentral.com/1471-2407/10/422/prepub
